# Evaluation and pilot testing of a multidisciplinary model of care to mainstream genomic testing for paediatric inborn errors of immunity

**DOI:** 10.1038/s41431-023-01321-z

**Published:** 2023-03-03

**Authors:** Tatiane Yanes, Anna Sullivan, Pasquale Barbaro, Kristian Brion, Georgina Hollway, Jane Peake, Peter McNaughton

**Affiliations:** 1 Queensland Paediatric Immunology and Allergy Service, Children’s Health Queensland, Brisbane, QLD 4101 Australia; 2 The Frazer Institute, Dermatology Research Centre, The University of Queensland, Brisbane, QLD 4102 Australia; 3 Queensland Paediatric Haematology Service, Queensland Children’s Hospital, Brisbane, QLD 4101 Australia; 4 Queensland Children’s Hospital Laboratory, Pathology Queensland, South Brisbane, QLD 4101 Australia; 5 Department of Molecular Genetics, Pathology Queensland, Royal Brisbane and Women’s Hospital, Brisbane, QLD 4029 Australia; 6 Department of Paediatrics and Child Health, The University of Queensland, Brisbane, QLD 4072 Australia

**Keywords:** Genetic services, Immunological disorders

## Abstract

Molecular diagnosis of paediatric inborn errors of immunity (IEI) influences management decisions and alters clinical outcomes, through early use of targeted and curative therapies. The increasing demand for genetic services has resulted in growing waitlists and delayed access to vital genomic testing. To address this issue, the Queensland Paediatric Immunology and Allergy Service, Australia, developed and evaluated a mainstreaming model of care to support point-of-care genomic testing for paediatric IEI. Key features of the model of care included a genetic counsellor embedded in the department, state-wide multidisciplinary team meetings, and variant prioritisation meetings to review whole exome sequencing (WES) data. Of the 62 children presented at the MDT, 43 proceeded to WES, of which nine (21%) received a confirmed molecular diagnosis. Changes to treatment and management were reported for all children with a positive result, including curative hematopoietic stem cell transplantation (*n* = 4). Four children were also referred for further investigations of variants of uncertain significance or additional testing due to ongoing suspicion of genetic cause after negative result. Demonstrating engagement with the model of care, 45% of the patients were from regional areas and on average, 14 healthcare providers attended the state-wide multidisciplinary team meetings. Parents demonstrated understanding of the implications of testing, reported minimal decisional regret post-test, and identified benefits to genomic testing. Overall, our program demonstrated the feasibility of a mainstreaming model of care for paediatric IEI, improved access to genomic testing, facilitated treatment decision-making, and was acceptable to parents and clinicians alike.

## Introduction

Inborn errors of immunity (IEI) are a group of severe inherited genetic disorders, causing recurrent infections, malignancies, allergies, and autoinflammation. Collectively, the prevalence estimates are 1 in ~1000 to 5000 births [1]. In the paediatric setting, IEI represents a serious illness, often associated with severe and life-limiting complications. These patients often display a heterogenous phenotype with overlapping immunologic, haematologic, rheumatologic, and malignant features, resulting in diagnostic delays [2]. Currently, over 450 single genes are known to be associated with IEI [3], and early molecular diagnosis of IEI improves health outcomes, reduces health expenditure, and mitigates psychological distress in families [4–6]. Furthermore, a genetic diagnosis can result in curative treatment, in the form of hematopoietic stem-cell transplantation (HSCT), which when required, is best performed early in the disease course prior to onset of significant end-organ damage [4]. Consequently, genomic testing has become essential in the diagnosis and management of children with IEI [7]. Currently, whole exome sequencing (WES) is the most cost-effective approach for genetic diagnosis of IEI [8, 9].

Similarly to most European countries, provision of genomic testing in Australia is primarily overseen by publicly funded clinical genetic departments, which are typically staffed by clinical geneticist and genetic counsellors [10]. In Queensland, Australia, provision of genomic testing within the public healthcare system is overseen by a state-wide clinical genetics service. This model of care (MoC) provides centralised clinical genetic services, covering diagnostics, genetic counselling, and management advice to individuals and families with a genetic condition. In line with national and international trends, referrals and use of genetic services in Queensland has grown significantly (volume of genetic testing has increased by 47% since 2012) [11]. This demand is expected to continue growing with the ever-increasing number of genetic tests available, reduction in costs of testing, and greater awareness of the value of genetic diagnosis [12].

New MoCs have recently been developed to address the growing demand for genetic services. Mainstreaming is the provision of genomic testing within routine clinical practice, such as an immunology outpatient clinic [13]. Several mainstreaming MoCs have been reported, including provision of genetic testing for cancer risk by non-genetic healthcare providers, with referrals to genetic services if the patient is found to have a positive result [14]. Genetic counsellors have also been embedded in non-genetic health services to facilitate mainstreaming [15, 16]. In Australia, a genetic counsellor embedded MoC for adults with hereditary cancer has been shown to be cost-effective, and associated with improved clinical outcomes, including increased identification of individuals eligible for genetic testing, reduced time to testing and results, and changes to patient management [13, 17]. Improved efficacy of referrals was also reported, with >400 unnecessary appointments saved from the genetic services, and improved referrals for those with positive results and family members for predictive testing [13, 17]. Internationally, a mainstreaming MoC for children with bone marrow failure has resulted in improved access to diagnostic genomic testing and information to facilitate clinical decisions [18].

Implementation of genomic testing is a complex process, requiring a whole system approach [19]. Beyond the laboratory, genomic testing requires upstream and downstream services such as identification of eligible patients, selection of gene lists, genetic counselling, interpretation of complex genomic testing results and clinical decision-making [19, 20]. Central to this process is patient-centred care to ensure families have access to appropriate counselling and support to explore the ethical, legal, and social implications of genomic testing [20]. Similarly, workforce training is required with non-genetic healthcare providers reporting limited knowledge and comfort accessing genomic testing [21, 22]. As such, development of mainstreaming MoC for genomic testing should address known barriers to facilitate uptake of testing by clinicians and patients alike, while mitigating the potential for adverse health and psychosocial outcomes.

The Queensland Paediatric Immunology and Allergy Service (QPIAS), based at the Queensland Children Hospital and Health Service (QCHHS), Brisbane, Australia, provides state-wide, publicly funded paediatric immunology care, including overseeing inpatient and outpatient clinics. The department is staffed by a team of paediatric immunologists and nursing staff. Historically, patients identified by the QPIAS team for a potential IEI diagnosis were referred to the state-wide clinical genetic service for assessment. However, given the increasing demand for genetic services, a mainstreaming MoC for paediatric IEI was developed and pilot tested to facilitate the provision of genomic testing. The present study aimed to evaluate:

i. The feasibility and efficacy of mainstreaming MoC in identifying positive cases of IEI,
ii. the impact of genomic testing on treatment outcomes, and
iii. the patient-reported outcomes of parents of children who had genomic testing.

## Methods

### Study design and MoC development

A prospective cohort study was developed to evaluate the feasibility, diagnostic rate, and patient-reported outcomes associated with mainstreaming genomic testing for IEI. A multidisciplinary team was established, comprised of paediatric immunologists (PM and JP), paediatric haematologist (PB), genomics scientist (KB, GH), genetic counsellor (TY), and clinical nurse consultant (AS). The MoC was informed by the literature and aimed to address known barriers to genomic implementation, including minimising misuse and misinterpretation of genomic testing, upskilling non-genetic healthcare clinicians, and providing appropriate genetic counselling.

**Fig. 1:**
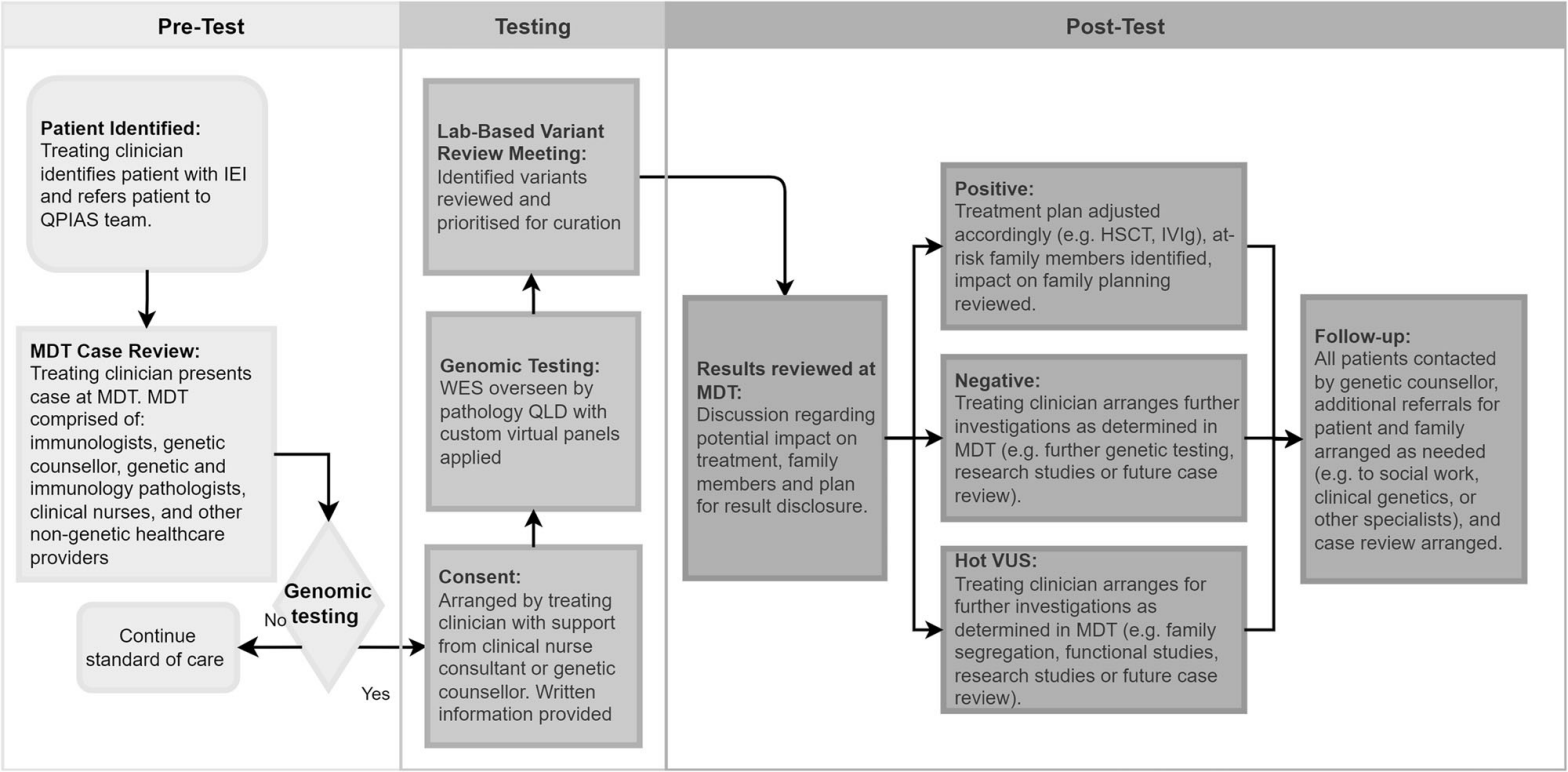
Inborn errors of immunity model of care at the Queensland Children’s Hospital and Health Service. The model of care is comprised of three phases: (i) pre-test includes MDT, case review, and selection of testing modality and gene lists, (ii) testing whereby informed consent is obtained, and lab-based variant review meeting conducted, and (iii) results reviewed at MDT and management outcome based on genomic testing results (i.e., positive, negative, VUS). HSCT haematopoietic stem cell transplantation, IEI inborn errors of immunity, IVIg intravenous immune globulin, MDT multidisciplinary team meeting, QLD Queensland, Australia, QPIAS Queensland Paediatric Immunology and Allergy Services, WES whole exome sequencing.

The final MoC was comprised of a fortnightly multidisciplinary team meeting to review all new cases for suitability for genomic testing and virtual panel selection (Fig. 1). Singleton WES was conducted for all participants, with sequencing and analysis provided by Pathology Queensland. Informed by the International Union of Immunological Societies (IUIS) Expert Committee [3], PanelApp Genomics England, (green, diagnostic-grade genes), and Queensland Health expert clinicians, 21 different virtual panels were developed (Appendix 1).

Following sequencing, secondary bioinformatics, and variant filtration, cases were discussed at a variant prioritisation meeting (VPM), which involved reviewing variants identified from the WES, and consensus agreement between clinicians and the genomic scientist on variants for further evaluation. Only variants relevant to the patient’s presentation were curated for pathogenicity. This clinically driven approach outperforms purely computational tools, and is associated with reduced turnaround time for variant curation [23]. Selected variants were classified according to ACMG Guidelines, ACGS Best Practice Guidelines, and SVI recommendations using evidence available at the time of reporting [24–26]. Results were then discussed at lab-based variant review meetings prior to reporting. All results were reviewed at the MDT with discussion regarding potential impact on treatment, additional investigations required, identification of at-risk family members, and required referrals (Fig. 1). To promote the MoC and support engagement across the health service, mailing lists were developed that included heads of departments from QCHHS with updates about the study and MDT agendas. Open invitations for clinicians with interest in paediatric IEI to attend the MDT were also distributed and the team conducted various presentations, including at departmental meetings and state-wide paediatric grand rounds.

### Participant eligibility and recruitment

Eligible participants included children, under the age of 18 years, referred to the QCHHS with a suspected monogenic IEI. Children were eligible to participate in the study regardless of geographical location in Queensland, and whether they were receiving treatment as an inpatient or outpatient. Given the heterogenous nature of IEI, children were eligible from across the health service, and there were no exclusions regarding referring department. Eligible children were identified by their treating clinician, who would then refer the patient to the QPIAS team and present the case at the MDT (Fig. 1). Participants were excluded if they had a confirmed genetic diagnosis of IEI. Informed consent for the study and WES was sought from all parents or guardian of children with an initial discussion by the treating clinician. All families were then seen by the study clinical nurse consultant (AS) or genetic counsellor (TY) to review the genomic testing consent form and address any remaining questions about testing. All participants were provided with written information about the study and genomic testing at consent. Consent process included a discussion about the potential outcomes of testing (i.e., positive and negative results, variant of uncertain significance (VUS), and implications for family members). Data sharing, potential insurance implications, and future research options were also discussed. Participants were also informed there was a small chance of actionable incidental findings from WES, which would be reported if identified. However, potential incidental findings were mitigated through VPMs to include/excluded variants from curation. Recruitment occurred from between December 2020 until June 2021.

### Model evaluation

Feasibility and evaluation of the MoC included descriptive statistics on number of children undergoing WES, attendance at project MDT, and diagnostic rate. Among those with a positive result, impact on treatment outcome was recorded. Similarly, additional investigations were noted for those with a VUS or negative result.

### Patient-reported outcomes

Following consent, parents or guardians of children recruited to the study completed two surveys: pre- and post-test result. The survey was informed by the literature and included demographic variables, recall of consent discussion, satisfaction with consent process, hopes/expectations of genomic testing, and impact of result on reproductive decision-making. Recall of potential outcomes of testing were also assessed with true/false questions (i.e., testing could result in a genetic diagnosis for your child, no genetic diagnosis identified, a VUS, and potentially have implications for other family members). Two validated measures were included in the post-test survey: the Decisional Regret Scale (DRS) [27] and the Genomic Outcome Scale (GOS) [28]. The DRS was used to measure regret over the decision to have genomic testing. The scale is comprised of five items (rated from strongly disagree to strongly agree) and scores range from 0 to 100, with higher scores indicating greater regret over the decision [27]. The DRS scores were defined into three categories based on a prior study of parents experience with genomic testing: no decision regret (DRS score 0), mild decision regret (DRS score 1–25), and moderate to high decision regret (DRS score >25) [29]. The GOS is a six-item scale (rated from strongly disagree to strongly agree) assessing empowerment related to genomic healthcare. A total score is calculated by summing all items, with higher scores indicating higher empowerment [27].

## Results

Of the 62 individuals presented at the MDT, 44 were identified as suitable for genomic testing, of which 43 consented to participate and proceeded to WES. Reasons, why the 19 children were not recommended genomic testing through the MoC, included: incomplete phenotype information presented (*n* = 2), recommendation for additional investigations before genomic testing (*n* =  1), having had prior genetic testing and research pathway recommended instead (e.g., gene discovery or functional studies) (*n* = 5), low a priori diagnostic yield from genomic testing (*n* = 10), and referral to clinical genetics due to complex phenotype that included developmental delay (*n* = 1). No patient was presented twice at the MDT during the study timeframe. However, since the conclusion of the MoC evaluation, one child has been re-presented and proceeded with genomic testing.

Of the 43 children enrolled, 20 (47%) were females, and the mean age was 9 years (SD 5.2) (Table 1). Fourteen children (33%) were existing patients of QPIAS, for whom there had been ongoing suspicion of a genetic condition. Of the 14 children, seven previously had panel testing for genes related to IEI and had received negative results.

**Table 1:** Participant demographic and clinical characteristics.

Children Demographic Characteristics (*n* = 43)	*n* (%)	Parent/Careers Demographic Characteristics (*n* = 38)	*n* (%)
*Sex*		*Relationship to child*	
Female	20 (47)	Mother	29 (76)
Male	23 (53)	Father	7 (18)
*Age (years)*		Carer	2 (5)
<1	3 (7)	*Age (years)*	
1–5	10 (23)	<30	7 (19)
6–10	9 (21)	31–40	14 (38)
11–15	15 (35)	40–50	10 (27)
16–18	6 (14)	51–60	6 (16)
*Demographic location*		60+	0 (0)
Metropolitan	24 (56)	*Ancestry*	
Regional	19 (44)	European	23 (61)
*Referring department*		Asian	4 (11)
Immunology	21 (49)	Māori	4 (11)
Hematology	5 (12)	Aboriginal and Torres Strait Islander	2 (5)
Gastroenterology	5 (12)	Other	5 (13)
Rheumatology	6 (14)	*Employment*	
Oncology	5 (12)	Full time	14 (40)
Respiratory	1 (2)	Part time	9 (26)
*Reason for Referral*		Not employed	12 (34)
Inflammatory bowel disease	9	*Education*	
Combined immune deficiency	6	High school	11 (31)
Immune dysregulation	6	Certificate	10 (29)
Autoinflammation	6	University or higher	14 (40)
Severe aplastic anaemia/bone marrow failure	5		
Susceptibility to atypical infection	4		
Evans Syndrome	3		
Predominantly antibody deficiency	2		
Severe combined immune deficiency	1		
Hemophagocytic lymphohistiocytosis	1		
*Existing patient of QPIAS*			
Yes	14 (33)		
No	29 (67)		
*Results*			
Positive	9 (21)		
Negative	32 (74)		
VUS	2 (7)		

*QPIAS* Queensland Paediatric Immunology and Allergy Service.

### Engagement with MoC

Children were referred from various departments within QCHHS, namely immunology (*n* = 21), rheumatology (*n* = 6), haematology (*n* = 5), gastroenterology (*n* = 5), and oncology (*n* = 5) (Table 1). Of the 43 children, 24 were from metropolitan area (56%), and 19 from regional Queensland (44%). Over the course of the study, there were 22 MDTs, which on average were attended by 14 different healthcare professionals from across the state. In addition to the research team, the MDTs were attended by various paediatric clinicians (e.g., gastroenterologists, rheumatologists, oncologists, etc), adult immunologists, immunology pathologists, and nurses. Twenty-two VPMs were conducted.

### Model evaluation

Most common reasons for referrals were very early onset inflammatory bowel disease (*n* = 9), immune dysregulation (*n* = 6), combined immune deficiency (*n* = 6), and autoinflammation (*n* = 6) (Table 1). Mean time from referral to sample being sent to the laboratory was 60 days (range 0–215 days). Reasons for delayed sample send-away included time taken to arrange genomic testing consent, failure to attend appointments, and challenges arranging sample collection (e.g., sample collection and transfer at regional centres and blood collection for children with needle fear). The mean time from consent to WES result was 149 days (range: 50–270 days).

Of the 43 children who had WES, nine (21%) received a confirmed genetic diagnosis (Tables 1 and 2), of whom three were existing patients of the service. No genetic diagnosis was achieved for children referred for autoinflammation (*n* = 6), severe aplastic anaemia/bone marrow failure (*n* = 5), and predominantly antibody deficiency (*n* = 2). Two children with a negative result were referred for additional investigations due to ongoing suspicion of monogenic causes of IEI (Table 2). One child was referred for trio whole genome sequencing for a gene discovery study via the Clinical Immunogenomics Research Consortium Australasia (CIRCA) [30]. The second child was referred to the clinical genetics service for review and consideration of whole genome sequencing due to detection of three different heterozygous variants in the complement deficiency pathway (a likely pathogenic variant in *C5, C6*, and *C7*). Complement deficiencies are more commonly associated with recessive inheritance, thus, further investigation of a second pathogenic variant was indicated (Table 2) [31]. VUS were reported in two children, with one referred for family segregation and functional studies, and another for ongoing research through CIRCA [30]. No changes to management were made for the 32 children with negative results, and all continued receiving standard of care. Negative and VUS cases received genetic counselling post-results as requested by the treating clinician or families. Reasons for genetic counselling request included discussing uncertainty of result, implications for families, and additional testing for VUS resolution. Consultations included discussion of ongoing risk of a genetic condition, implications for family planning (where possible, recurrence risk based on empirical data), and exploring uncertainty related to VUS.

**Table 2:** Diagnostic rate and outcome of genomic testing.

Primary IEI presentation	No. patients	Virtual Panel Results	Genes	Patient treatment and additional investigations
Inflammatory bowel disease	9	Positive (*n* = 1)	*XIAP*	HSCT
		Hot VUS (*n* = 1)	*PSTPIP1*	Inconsistent family segregation studies: variant no longer considered hot
Combined immune deficiency	6	Positive (*n* = 4)	*CD40L*	HSCT
			*IFNAR1*	IRT
			*CORO1A*	IRT
			*ATM*	Standard ataxia telangiectasia management plan
		Hot VUS (*n* = 1)	*CARD11*	Referred to national immunology consortia for additional VUS investigation
Immune dysregulation	6	Positive (*n* = 1)	*CFHR1-CFHR4*	Change in surveillance and early treatment with eculizumab if recurrence of disease
		Negative, ongoing investigation (*n* = 1)	Nil	Referred for WGS for gene discovery research
Susceptibility to atypical infection	4	Negative, ongoing investigation (*n* = 1)	3 heterozygous LPV reported each in: *C5*, *C6*, and *C7*	Referral to clinical WGS to identify if there is a second variant in any of the three candidate genes
Evans Syndrome	3	Positive (*n* = 1)	*NFKB1*	Changes in surveillance
Severe combined immune deficiency	1	Positive (*n* = 1)	*ADA*	HSCT
Hemophagocytic lymphohistiocytosis	1	Positive (*n* = 1)	*HAVCR2*	HSCT

Summary of the 13 cases with a positive result (*n* = 9), hot VUS (*n* = 2), and negative result for further investigation (*n* = 2).

*HSCT* hematopoietic stem cells transplantation, *IRT* immunoglobulin replacement therapy, *VUS* variant of uncertain significance, *LPV* likely pathogenic, *PV* pathogenic. *Het*. heterozygous. *Hom* Homozygous.

Changes to treatment and management were reported among all nine children with a positive result, including: curative HSCT (*n* = 4), immunoglobulin replacement therapy (IRT) (n = 2), and changes in surveillance (*n* = 3) (Table 2). Mean age at time of HSCT was 6 years (range 6 months to 16 years). All positive cases received genetic counselling in relation to understanding the genetic testing results, coping with a genetic diagnosis, and family planning options. Referrals to the state-wide clinical genetic service were also facilitated as needed for family carrier testing.

### Patient reported outcomes

**Fig. 2:**
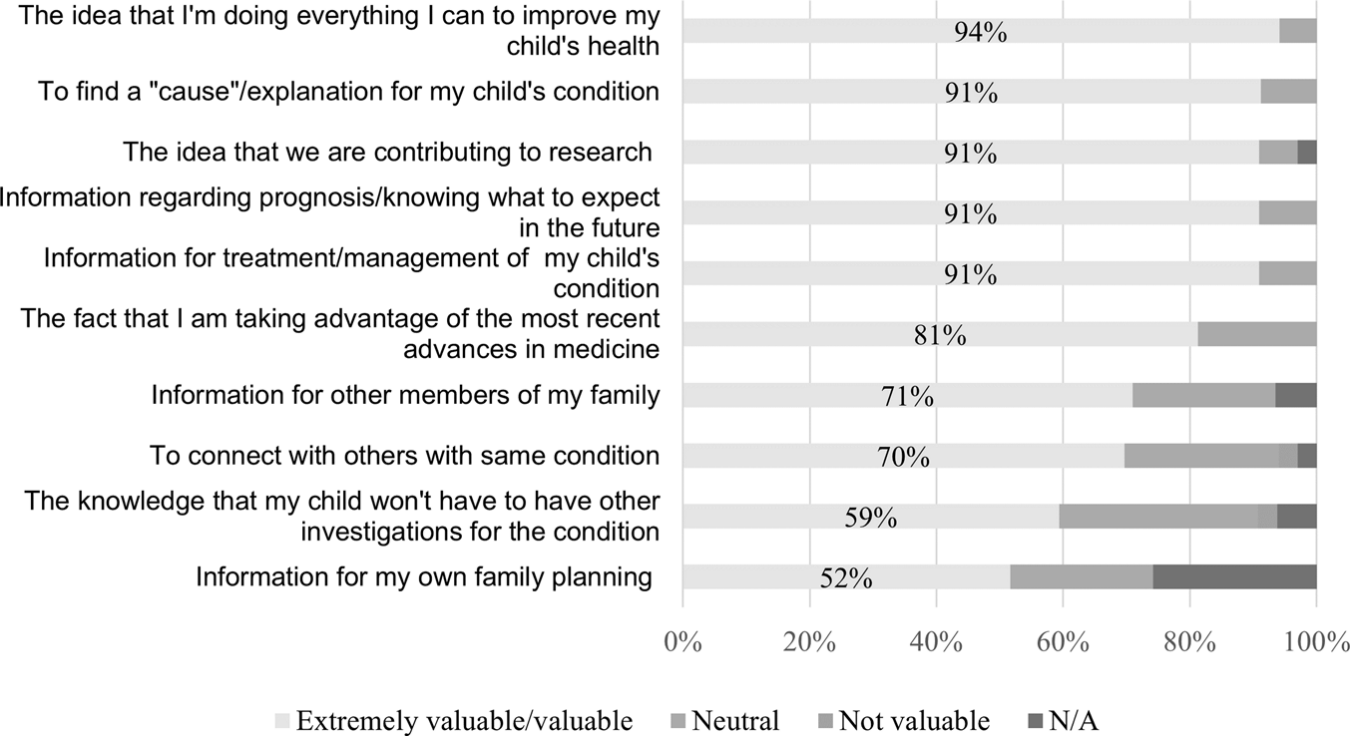
Parents/guardians hope and motivations for consenting for genomic testing for their child before testing. Data indicates parents/guardians’ level of agreement regarding their hopes and motivations for consent for genomic testing for their children. Parents/guardians most commonly rated items related to seeking answers for their child’s illness and informing their treatment/management.

Of the 43 children enrolled in the study, 38 (88%) parents or guardians completed the pre-test survey and 20 (47%) the post-test survey. Surveys were more frequently completed by mothers (*n* = 29, 76%) and the mean age of parents/guardians was 39 years (SD 9.1) (Table 1). Most reported European ancestry (*n* = 23, 61%), followed by Asian (*n* = 4, 11%), and Māori (*n* = 4, 11%). When asked to recall the consent process, the majority recalled a discussion about genomic testing with a clinician (*n* = 34, 94%) and most described this conversation as extremely valuable or valuable (*n* = 30, 94%) (Table 3). Less parents recalled receiving written information, including a copy of the consent form and genomic testing fact sheet (*n* = 14, 40%). Of those who recalled receiving written information, most described this as extremely valuable or valuable (*n* = 19, 82%). Of the four parents who did not recall a verbal discussion with a clinician, all recalled receiving written information about the genomic test. Most participants also felt they had received enough information to make an informed decision regarding their child’s genomic testing (*n* = 33, 92%), that they had opportunities to ask questions (*n* = 35, 97%), and they did not have any remaining concerns about the test (*n* = 31, 86%). When asked about the possible outcomes of the test, most correctly identified as true that the genomic test could include: a genetic diagnosis for their child (*n* = 30, 83%), no genetic diagnosis identified (*n* = 25, 69%), a VUS (*n* = 21, 58%), and potentially have implications for other family members (*n* = 25, 69%). No parent incorrectly answered these questions as false. However, several participants indicated they did not know the potential outcomes of testing: positive indicating a genetic diagnosis for their child (*n* = 6; 17%), no genetic diagnosis (*n* = 11, 31%), VUS (*n* = 15, 42%), and implications for family members (*n* = 11, 31%). Lastly, when asked to rate their reasons for having genomic testing, most rated as extremely valuable/valuable: “*the belief that they were doing everything they could to improve their child’s health”* (*n* = 31, 94%), and *“to find a cause for my child’s condition”* (*n* = 31, 91%) (Fig. 2).

**Table 3:** Frequency of agreement with pre-and post- genomic testing survey amongst parents of children who had genomic testing.

Item	*n* (%)
*Pre-Test (n* = *38)*	
*Recall of consent process*	
Conversation with clinician	34 (89)
Receiving printed information	14 (37)
Don’t remember	0 (0)
*Did you receive enough information to make an informed decision about genomic test for your child?*	
Yes	33 (92)
No	0 (0)
Unsure	3 (8)
*Were you given enough opportunity to ask questions about the* genomic *test for you child?*	
Yes	35 (97)
No	1 (3)
Unsure	0 (0)
*Did you have any* remaining *concerns about your child’s genomic test?*	
Yes	1 (3)
No	31 (86)
Unsure	4 (11)
*Correctly identified possible results from genomic testing*	
Positive	30 (58)
No genetic diagnosis is identified	25 (69)
Variant of uncertain significance	21 (58)
Have implications for family	25 (69)
*Post-Test (n* = *20)*	
*Impact on of genetic testing on reproductive decision*	
*No impact*	14 (82)
*More children*	2 (12)
*Less children*	1 (6)
*Decision regret scale scores*	
No regret (score = 0)	9 (53)
Mild regret (1–25)	7 (41)
Moderate to high regret (>25)	1 (6)
*Genomic outcome scale*	
<10	1 (8)
11–20	6 (50)
20–30	5 (42)

In the post-result surveys, all but one participant correctly recalled their child’s genomic test results (*n* = 19, 95%). The remaining participant indicated they could not recall that their child had received a negative result. Most participants (*n* = 19, 95%) reported their child’s result was not unexpected. One parent reported they had not expected a negative result and described that they “*still don't have answers*”. All parents reported they received enough information to understand their child’s result. In relation to reproductive decisions, most parents indicated their child’s result did not impact their decision whether to have more children (*n* = 14, 82%) (Table 3). Of the three participants who indicated the results impacted their reproductive decisions, one received a negative result and indicated they were considering having less children due to the uncertainty related to recurrence risk, one received a *de novo* positive result and indicated they were uncertain about recurrence risk, and one with a positive result indicated they would now consider using preimplantation genetic diagnosis.

Due to the small sample size, it was not possible to evaluate the impact of genomic test results on decisional regret and empowerment (Table 3). Half of the participants (*n* = 9, 53%) indicated no regret over their decision to consent for genomic testing for their child. Of the remaining participants, eight (41%) had mild regret (scores 5–25), and one (6%), whose child received a positive result, indicated moderate/high regret (score = 43) (total DRC M 7.1, SD 11.4, range 0–43). The participant with moderate regret did not provide any response in the open text box to indicate reasons for their regret. The highest empowerment scores were all reported by parents of children who received a positive result (range 30-20) (total GOS M 19.2, SD 7.0, range 8–30). The lowest scores were reported by parents of children with a negative result (range 20-8) and VUS (both scores = 12).

## Discussion

Genomic testing has increasingly become an integral part of the diagnosis and management of children with IEI. This pilot study demonstrated the feasibility and clinical utility of a mainstreaming MoC for genomic testing for children with IEI in Queensland, Australia. Over the course of the study, 43 children underwent genomic testing, and the diagnostic rate was 21%. A positive result was associated with changes to health management and treatment for all children, including IRT and curative HSCT. There was high engagement with the MoC as demonstrated by referral numbers and participation in the MDT by clinicians from across the state. However, few participants were Aboriginal and Torres Strait Islander or from culturally and linguistically diverse populations. Parents also reported minimal decisional regret regarding their decision to consent for genomic testing for their child. However, there were some knowledge gaps in relation to the potential outcomes of genomic testing.

The diagnostic yield of 21% is consistent with previous studies reporting outcomes from genomic testing in patients with IEI [32, 33]. Conclusive molecular diagnoses resulted in clinically important changes including targeted therapies and curative HSCT for patients with X-linked lymphoproliferative syndrome (*XIAP*), X-linked hyper IgM syndrome (*CD40L*), severe combined immune deficiency (*ADA*) and subcutaneous panniculitis-like T-cell lymphoma (*HAVCR2*). In patients where a molecular diagnosis was not identified, clinicians were able to continue with established treatment pathways with reassurance that there was not a superior treatment available for their patient. Although our study did not include cost-analysis, it is well-reported that early molecular diagnosis of IEI is cost-effective and is associated with improved health outcomes [4, 6]. Cost benefits are common due to a reduction in hospitalisation time, provision of curative HSCT, reduced mortality, and impact on productivity such as less missed school and workdays [4].

Our MoC aimed to facilitate prompt access to genomic testing for paediatric IEI, while addressing the known complexities and issues associated with mainstreaming [19, 20]. The inclusion of an MDT in our MoC provided the opportunity for peer review and ensured that genomic testing was appropriately ordered, and results correctly interpreted. In some cases, the MDT provided support for clinicians in not proceeding with testing when there was consensus of low a diagnostic yield. A key limitation of our MoC was the longer-than-anticipated turnaround time for reporting of results through the in-house pathology laboratory. Such delays in testing turnaround time can impact access to early treatment and diagnosis of patients. Since completion of the study, strategies have been implemented to address testing turnaround time, including increasing workforce capacity, and WES results are being now being reported within two to three months. However, further increases in variant curation workforce is still required to meet growing demand for genomic testing. Our MoC was also limited to singleton WES due to funding constraints, and limited processes to arrange parental samples and consent for all parents/guardians. Compared to singleton testing, trio WES is associated with increased clinical utility such as higher diagnostic rate, cost-effectiveness, and reduced clinician and scientist variant curation time [34]. Since completion of the pilot study, increased access to trio testing is being implemented that includes updated costings.

A unique aspect of our MoC was embedding a genetic counsellor within the paediatric immunology department. Our findings are in line with previous studies involving MoC with embedded genetic counsellors, including reduced time accessing testing and result, changes to patient management, and reduced number of appointments required for patients to access genetic testing [13, 17]. Anecdotally, the collaborative approach between the treating specialists and genetic counsellor facilitated mutual learning across specialties and supported a co-ordinated approach to patient care and use of genomic information [30]. Thus, developing stronger expertise of the diagnostic, therapeutic and implications of genomics testing in paediatric IEI across QCHHS. It is anticipated that our MoC can be used to support mainstreaming of genomic testing across other settings with similar burdens of genetic conditions, such as metabolic and neurology. Increasing genetic counselling workforce capacity will be needed to support widespread mainstreaming of genomic testing, with non-genetic healthcare providers frequently reporting a lack of genetic knowledge and time pressure to perform duties related to genomic testing (such as obtaining informed consent for genomic testing, interpreting, and disclosing complicated results, and identifying the familial, ethical, legal and social implication of genomic testing) [22, 35]. Similarly, consideration will be needed of the challenges experienced by genetic counsellors in mainstreaming services, including limited understanding of the role among colleagues and managers, support for career progression, and feelings of isolation [10].

Defining informed consent for genomic testing has long been acknowledged as a challenging concept given the broad and uncertain nature of potential outcomes [36]. Evaluation of the MoC identified that most parents/guardians, recalled a discussion about genomic testing with a clinician, felt they had received enough information to make an informed decision about their child’s test, and had no/minimal regret over their testing decision. However, when asked about potential outcomes of testing, there were knowledge gaps identified, including 31% being unaware the test could have implications for family members, 17% did not know testing could result in a genetic diagnosis for their child, and less than half recalled receiving written information. Studies exploring parents’ decision-making about genomic testing in paediatric care have identified that despite parents’ vulnerability, most reported minimal regret about their decision to undergo genomic testing and identified benefits to testing [29, 37, 38]. However, some parents reported feeling that their decision to consent for testing was rushed and that additional support was needed [29, 37, 38]. Combined, these finding highlights the challenges in discussing informed consent, particularly in paediatric mainstreaming setting where parents may be coping with stressors from their child’s health condition. Future work on the MoC should consider options to improve the consent process, such as implementation of dynamic consent and patient-friendly videos and fact sheets [36, 39].

Although it was not possible to evaluate impact of genomic test result on empowerment, parents of children with a positive result reported the highest scores for this measure. This result suggests that a genetic diagnosis may improve parents’ sense of empowerment including cognitive control, decisional control, behavioural control, emotional regulation, and hope [28]. Future studies should aim to further explore parents’ experiences with genomic testing, including longer-term follow-up with validated measures across multiple timepoints.

Our MoC and project evaluation is not without limitations. Firstly, our MoC is time and cost-intensive, requiring support from various experts from across the genomic testing pipeline and funding for costly genomic testing. Nevertheless, our MoC is in line with similar genomic MoCs in Australia [40], and we hypothesise that this process is cost-effective through the early diagnosis of IEI and implementation of interventions and curative treatments. We also have a small sample size and limited long-term data on clinical and psychosocial outcomes associated with genomic testing. Our MoC was not inclusive of diverse populations. In Australia, access disparities to health services are well reported for Aboriginal and Torres Strait Islander people and those from culturally and linguistically diverse populations [41, 42]. Disparities in service access are complex, and largely driven by macro-and micro-level issues, such as lack of infrastructural support, standardized practice, and individual challenges (e.g., lack of trust, fear, rapport, and difficulties in navigating health systems) [41, 42]. While it was beyond this project to address these issues, future work should aim to address access disparities including developing separate pathways for diverse populations, further implementation of care co-ordinators, and improved education for referrers to identify patients eligible for testing. Lastly, our pre-test survey did not include a measure of empowerment and it is not known what impact the genomic test result had on this outcome. Despite these limitations, our study achieved a diagnostic yield that is consistent with prior literature and demonstrated the feasibility of a mainstreaming MoC for paediatric IEI, which supported prompt access to genomic testing and was well received by patients and clinicians alike.

## Data Availability

The datasets generated during and/or analysed during the current study are available from the corresponding author on reasonable request.
